# Functional Update of the Auxiliary Proteins PsbW, PsbY, HCF136, PsbN, TerC and ALB3 in Maintenance and Assembly of PSII

**DOI:** 10.3389/fpls.2016.00423

**Published:** 2016-04-07

**Authors:** Magdalena Plöchinger, Serena Schwenkert, Lotta von Sydow, Wolfgang P. Schröder, Jörg Meurer

**Affiliations:** ^1^Department Biologie I, Molekularbiologie der Pflanzen (Botanik), Ludwig-Maximilians-UniversitätPlanegg-Martinsried, Germany; ^2^Department Biologie I, Biochemie und Physiologie der Pflanzen, Ludwig-Maximilians-UniversitätPlanegg-Martinsried, Germany; ^3^Umeå Plant Science Center and Department of Chemistry, Umeå UniversityUmeå, Sweden

**Keywords:** PSII photosystem II, cytochrome b559, assembly, low molecular mass proteins, *Arabidopsis*

## Abstract

Assembly of Photosystem (PS) II in plants has turned out to be a highly complex process which, at least in part, occurs in a sequential order and requires many more auxiliary proteins than subunits present in the complex. Owing to the high evolutionary conservation of the subunit composition and the three-dimensional structure of the PSII complex, most plant factors involved in the biogenesis of PSII originated from cyanobacteria and only rarely evolved *de novo*. Furthermore, in chloroplasts the initial assembly steps occur in the non-appressed stroma lamellae, whereas the final assembly including the attachment of the major LHCII antenna proteins takes place in the grana regions. The stroma lamellae are also the place where part of PSII repair occurs, which very likely also involves assembly factors. In cyanobacteria initial PSII assembly also occurs in the thylakoid membrane, in so-called thylakoid centers, which are in contact with the plasma membrane. Here, we provide an update on the structures, localisations, topologies, functions, expression and interactions of the low molecular mass PSII subunits PsbY, PsbW and the auxiliary factors HCF136, PsbN, TerC and ALB3, assisting in PSII complex assembly and protein insertion into the thylakoid membrane.

## Introduction

Photosynthesis converts sunlight energy into chemical energy and takes place in chloroplasts and cyanobacteria. This process is fundamental for life on our planet and starts with the excitation of electrons in the multi-subunit and pigment-containing photosystem (PS) II complex. PSII has a molecular mass of more than 600 kDa and comprises more than 30 protein subunits, pigments and cofactors, such as Ca^2+^, Cl^-^ and different oxidation states of Fe and Mn. Our current knowledge about the function, structure and components of PSII is quite comprehensive, yet still little is known about how the complex assembles or disassembles for repair and even less regarding how its function is fine-tuned for optimal performance under fast and ever changing light and temperature conditions as well as varying water availability. For optimal flexibility the PSII complex requires a set of auxiliary proteins which assist in quenching of excited states, electron flow, assembly, repair and stability. Previous genetic and biochemical work provided insights into these processes. So far, more than 30 auxiliary (see recent reviews; Shi et al., 2012; Nickelsen and Rengstl, 2013) components have been identified that bind at least transiently to the complex by interacting with individual proteins and/or protein sub complexes to optimize PSII function and assembly.

In this review we present an update on the latest findings of two possible structural and four auxiliary proteins which we have been studying. Information about the proteins is summarized in **Table 1**. PsbW is required for the PSII homodimerization and the function of the PsbY proteins still remains to be solved. PsbN and HCF136 are required for heterodimerization of PSII reaction center in the stroma lamellae, whereas TerC and ALB3 act on translation and/or incorporation of proteins into PSII and other thylakoid membrane complexes.

**Table 1:** Summary of auxiliary proteins associated with PS II discussed in this update.

Protein	Other designations	Gene *Arabidopsis*	Size (kDa) *Arabidopsis*	Location	Function
PsbW	T6B20.8	At2g30570	6.1	Grana, thylakoid membrane	PSII supracomplex formation
PsbY1/PsbY2	*YCF32*	At1g67740	4.7/4.9	PSII complex	Unclear at present
PsbN	*ORF43*	AtC00700	4.7	Stroma lamellae	heterodimeric PSII RC assembly
HCF136	*YCF48*	At5g23120	37	Thylakoid lumen	heterodimeric PSII RC assembly
TerC	PDE149 ATTERC	At5g12130	42	Integral thylakoid protein	Insertion of thylakoid membrane proteins
ALB3	ALBINO3	At2g28800	44,5	Integral thylakoid protein	Insertion of thylakoid membrane proteins


## PsbW

### Discovery, Localization and Topology of PsbW

The nuclear encoded PsbW protein was first discovered in spinach chloroplasts and found to contain a long bipartite transit peptide directing it to the thylakoid lumen (Lorković et al., 1995). The mature PsbW protein (formerly 6.1 kDa protein) is composed of 54 amino acids and predicted to have one transmembrane α-helix (Lorković et al., 1995; Hagman et al., 1997). The PsbW protein has been found to be highly conserved in algae such as *Chlamydomonas reinhardtii* (Bishop et al., 1999) and higher plants, but is absent in cyanobacteria (see Shi and Schröder, 2004 for a discussion on this). The localization of the PsbW protein within the PSII complex has not been unambiguously determined. The original analysis (Lorković et al., 1995; Hagman et al., 1997) placed the protein in or close to the PSII reaction center. Meanwhile, two other studies (Rokka et al., 2005; Granvogl et al., 2008) propose a connection to the Lhcb proteins. As the protein is absent in cyanobacteria the present crystal structures of PSII cannot give any suggestions to where the protein is located and the solution to this question has to await high resolution crystal structures from higher plants or algae.

### Functional Aspects of the PsbW Protein

The PsbW protein has several unique features which point to an important function in PSII. Firstly, it was shown that the PsbW protein is degraded under photoinhibitory conditions, i.e., high light treatments, to the same extent as the D1 protein of the PSII reaction center (Hagman et al., 1997). *Arabidopsis thaliana* (hereafter *Arabidopsis*) plants with reduced amounts of PsbW showed decreased levels of PSII core proteins and functional PSII complexes (Shi et al., 2000). Using *in vitro* translated PsbW protein in combination with BN-PAGE it was shown that the newly imported PsbW protein was assembled rapidly into dimeric PSII supercomplexes and that the negatively charged N-terminus of PsbW was crucial for this, as a recombinant form with an uncharged N-terminus did not incorporate into PSII (Thidholm et al., 2002). These findings were further corroborated using T-DNA insertion knock-out PsbW *Arabidopsis* plants (García-Cerdán et al., 2011). It was shown that the loss of PsbW destabilizes the supramolecular organization of PSII and no PSII-LHCII supercomplexes could be detected. The absence of “normal” PSII macroorganization leads to a decrease of the maximum PSII quantum yield, changed core protein phosphorylation and faster redox changes of the PQ pool (García-Cerdán et al., 2011). Thus, formation of the supramolecular organization of PSII seems to optimize the functions of the complex. Based on these analyses, it was suggested that PsbW is located close to the minor antennae of the PSII complex, which would explain that the PsbW protein is associated with the PSII reaction center and the LHC complex. Presumably, the PsbW protein is important for the assembly and/or stability of the PSII-LHCII supercomplexes in the grana regions of the thylakoid membrane.

## PsbY

### Discovery, Localization and Topology of PsbY

The PsbY protein was originally isolated and reported to be associated with PSII in tobacco and spinach (Gau et al., 1995). It is thought to exhibit a manganese requiring L-arginine metabolizing activity. Genomic inspections and alignments of the PsbY amino acid sequence revealed that the protein is present in all photosynthetic plants, in some algal chloroplasts (formerly *ycf32*) and also in various cyanobacteria (Sml0007) (Gau et al., 1998). In higher plants, PsbY is nuclear encoded and targeted to the thylakoids. The unique locus encodes two homologous products (PsbY-1 and PsbY-2) with molecular masses of 4.7 and 4.9 kDa, respectively. *In vitro* protein import analysis supports these predictions (Mant and Robinson, 1998; Thompson et al., 1999). The gene fusion is most probably a result of an intragenic duplication which occurred during endosymbiosis as in cyanobacterial species only one *psbY* gene exists. Immunological studies using a peptide specific antibody directed against the C-terminal part of PsbY-2 (NILQPALNQINKMRSGD) failed to identify a protein with a molecular mass of around 10 kDa corresponding to a protein fusion including both PsbY-1 and PsbY-2. Instead a single band of approximately 5 kDa was detected, corresponding to a cleaved PsbY product. Mass spectrometry in barley (*Hordeum vulgare*) revealed that PsbY-1 was absent in etioplasts and chloroplasts, while PsbY-2 appeared with high signal intensity (Plöscher et al., 2009). Taking all this together, PsbY-2 also seems to be the major product in barley and probably also in *Arabidopsis*.

### Functional Aspects of the PsbY Protein in Photosystem II

**FIGURE 1:**
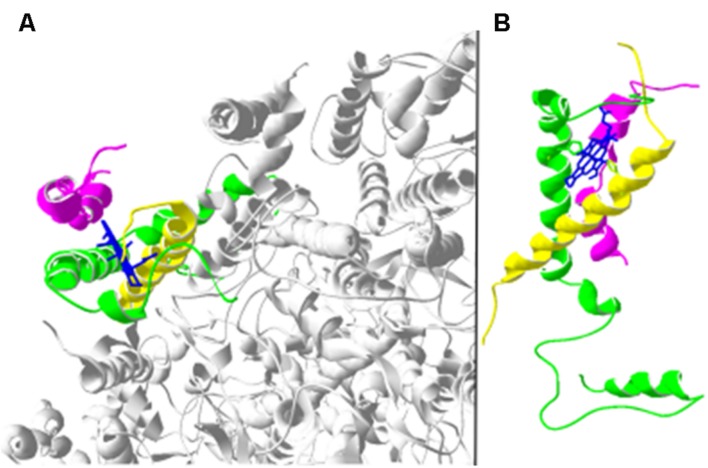
**Structure of the PSII complex from *T. vulcanus* (Pdb id: 4il6 [3]) generated by Swiss-pdb viewer program.** **(A)** Overview of PSII in the Cyt*b*_559_ area. Low molecular mass proteins: PsbE (green), PsbF (yellow) and PsbY (magenta). The heme group is shown in blue and PSII core in gray. **(B)** Close-up of PsbE, PsbF, and PsbY proteins showing ligands and the heme.

Co-expression analysis of the *psbY* gene showed that its expression is similar to other nuclear genes encoding PSII components and that it grouped close to genes encoding PsbO, PsbQ, PsbS and PsbTn, pointing toward a function in PSII (Shi et al., 2012). Interestingly, PsbY was missing in some of the early PSII structures (Ferreira et al., 2004; Kawakami et al., 2007). Furthermore, in the crystal structures of PSII from *Thermosynechococcus elongatus* the PsbY protein was located on the outside of the PSII complex in close connection with Cyt *b*_559_ (Zouni et al., 2001; Ferreira et al., 2004; Loll et al., 2005; Kawakami et al., 2007) and in close proximity to PsbE and PsbF, two low molecular mass proteins sharing a heme group and forming Cyt *b*_559_ (Guskov et al., 2009; Umena et al., 2011) (**Figure 1**). Thus, a direct interaction of PsbY with Cyt *b*_559_ seems possible. Cyt *b*_559_ is suggested to take part in the cyclic electron flow around PSII together with pheophytin, Q_A_ and Q_B,_ in order to protect PSII against photoinhibition (Barber and De Las Rivas, 1993; Kropacheva et al., 2003). Cyt *b*_559_ is found in three redox potential forms: low potential (LP), intermediate potential (IP), and high potential (HP). Interconversion between them is believed to be important for the photoprotection of the complex. However, the exact mechanism by which Cyt *b*_559_ is switching between the three potential forms is unclear at present. A number of possibilities for the interconversions were discussed, among them a change in the orientation of the axial histidine ligands (histidine residues from PsbE and PsbF) and a change in the polarity of the heme environment or in the coordination of the heme (Kaminskaya et al., 2007). An oxygen reductase activity (LP), superoxide oxidase activity (IP to HP), superoxide reductase activity (HP to IP) and plastoquinol oxidase activity (HP) were discussed to be involved in the different forms or conversions (Pospíšil, 2012). Indications of an additional quinone site apart from Q_A_ and Q_B_ was suggested (Kruk and Strzalka, 2001; Kaminskaya et al., 2007), which initially was believed to function as a switch for the different redox potential states of Cyt *b*_559_ (Kruk and Strzalka, 2001). Recently, this site was included in the crystal structure of PSII (Guskov et al., 2009; Lambreva et al., 2014), as the Q_C_ pocket between Cyt *b*_559_ and PsbJ. A detailed study of this quinone concluded that it does not have the characteristics needed to influence the redox potential of Cyt *b*_559_ (Guskov et al., 2009). The presence of an additional quinone, Q_D_, was also suggested (Kaminskaya and Shuvalov, 2013). However, Q_D_ has not been found in any of the numerous crystal structures presenting PSII, possibly due to a location outside of Cyt *b*_559_. The probability of Q_D_ residing in very close vicinity to PsbY is rather high and perhaps the evolutionary conserved tryptophan (Q109 and Q181 for *PsbY-1* and *PsbY-2*, respectively) could serve as a ligand for this quinone. Another possible function of PsbY is to stabilize the binding of PsbE and PsbF to the heme group by providing a shelter for Cyt *b*_559_ from oxidizing compounds.

## HCF136

### Expression, Localization and Structure of the HCF136 Protein

Unlike structural components of the photosynthetic apparatus, the HCF136 protein accumulates already in etiolated *Arabidopsis* seedlings, indicating an important role for HCF136 during the early stages of chloroplast development. Its expression level increases upon illumination in parallel with the appearance of constituent PSII subunits and the biogenesis of the first PSII complexes (Meurer et al., 1998). After its synthesis in the cytosol, plant HCF136 containing a bipartite transit sequence is imported into the chloroplast lumen by the Tat pathway (Meurer et al., 1998; Hynds et al., 2000). The mature HCF136 protein attaches to the stroma lamellae, the place where early PSII biogenesis and repair takes place. Therefore, a function of HCF136 as a constituent subunit of PSII could be excluded (Meurer et al., 1998). Association with the thylakoid membrane may occur via a conserved hydrophobic patch formed by 18 N-terminal amino acids and/or via interaction with other proteins. However, the rest of the lumenal protein is hydrophilic, harboring no predicted transmembrane domains (Meurer et al., 1998). The cyanobacterial HCF136 homolog named YCF (hypothetical chloroplast open reading frame) 48 is targeted to the thylakoid lumen in a Sec-dependent manner in *Synechocystis* sp. PCC 6803 (hereafter *Synechocystis)* (Hynds et al., 2000). Interestingly, a smaller amount of the protein resides in the PratA-defined membrane (PDM), a specialized membrane region of the thylakoids, where early steps of PSII biogenesis are thought to take place in cyanobacteria (Schottkowski et al., 2009; Rengstl et al., 2011).

**FIGURE 2:**
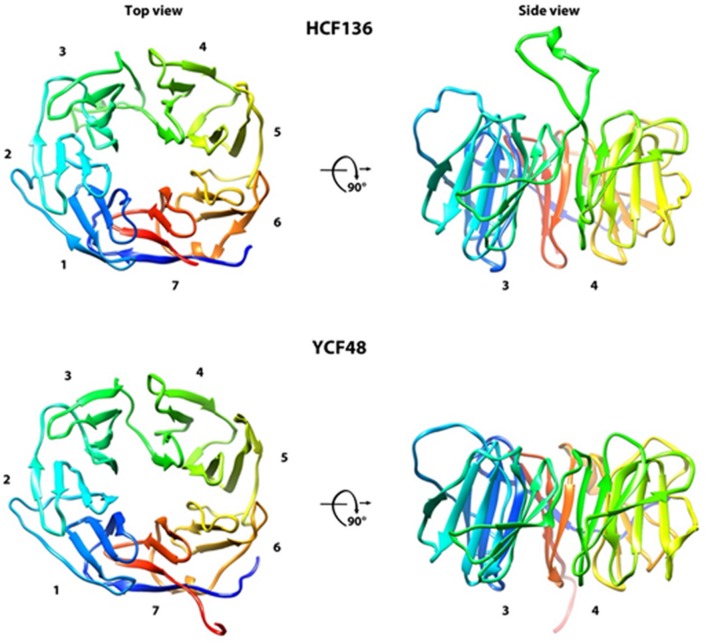
**Predicted structure of the mature HCF136 in chloroplasts and YCF48 in cyanobacteria.** The structure of HCF136 in *Arabidopsis* (C-score 0.81 ± 0.09) and *Thermosynechococcus elongatus* strain BP-1 (C-score 0.92 ± 0.06) was calculated by I-Tasser (Yang et al., 2015) and is shown from the top and the side view demonstrating the position of the additional loop in the chloroplast form.

The elucidation of the X-ray crystal structure of YCF48 from *Thermosynechococcus elongatus* revealed that the protein displays the shape of a β-propeller (Mabbitt et al., 2014) (deposited at http://pdbj.org/, PDB: 2XBG, Michoux et al., unpublished). It is built up by seven blade-shaped beta-sheets which are radially arranged, building up a central pore (Mabbitt et al., 2014) (**Figure 2**). Moreover, HCF136 of many eukaryotes is predicted to harbor an additional highly conserved internal stretch of 19 amino acids (Meurer et al., 1998) protruding between the 3rd and 4th blade of the propeller (**Figure 2**). Such propeller architecture has been reported to enable specific protein–protein interactions all around the formed funnel (Chen et al., 2011).

### Evolution and Gene Context of the Cyanobacterial HCF136 Homolog

In cyanobacteria, *YCF48* is often embedded into a gene cluster associated with PSII. It is located downstream of the rubredoxin *rubA* gene and upstream of the *psbE-F-L-J* gene cluster. RubA is associated with PSII core complexes and required for the accumulation of this complex (Wastl et al., 2000; Calderon et al., 2013). PsbE and PsbF encode for the two Cyt *b*_559_ subunits of PSII. PsbL and PsbJ also encode low-molecular-weight proteins of PSII which are required for stability and functional forward electron transport within PSII by affecting Q_B_ binding site properties (Ohad et al., 2004). The arrangement and composition of these genes is highly conserved in cyanobacteria, implying a possible functional correlation. Nevertheless, there are a few exceptions concerning the conserved arrangement of these genes: *YCF48* still resides next to *rubA* but is physically separated from the *psbE-F-L-J* gene cluster in *Synechococcus* sp. Ja-2-3B‘a, *Synechococcus* sp. Ja-3-3Ab and *Thermosynechococcus elongatus BP-1* whereas the arrangement has been subject to duplication and gene loss in *Acayochloris marina* (Calderon et al., 2013). Interestingly, no functional homologs of *YCF48* and *rubA* could be found in the recently discovered cyanobacteria UCYN-A which lacks PSII (Zehr et al., 2008). HCF136 is still encoded in the plastomes or cyanelles of the glaucocystophycean alga *Cyanophora paradoxa* upstream of the *psbE-F-L-J* gene cluster but was transferred into the nuclear genome most likely before divergence of the green and red lineages during endosymbiosis (Meurer et al., 1998). The HCF136 protein is present in all organisms performing oxygenic photosynthesis.

### HCF136/YCF48 Functions in Stabilization of pD1, Formation of the PSII Reaction Center and Presumably PSII Repair

A mutant screen based on the identification of *high chlorophyll fluorescence* (*hcf*) phenotypes led to the discovery of HCF136, the first identified assembly factor for PSII in plants (Meurer et al., 1998). Different from most knock outs of PSII assembly factors, homozygous *Arabidopsis hcf136* mutants were seedling lethal and could only survive on sucrose-containing medium, caused by their inability to accumulate PSII complexes. Consequently, they suffered from photooxidative stress even under very low light conditions and displayed a pale phenotype (Meurer et al., 1998). Transcription and translation of PSII genes and proteins in the mutant were comparable to the wild type, suggesting that PSII proteins are synthesized normally but are rapidly degraded (Meurer et al., 1998). As a secondary effect and depending on the light intensity, the mutants lacking HCF136 also accumulated lower amounts of PSI proteins; however, the PSI complexes present were functional (Plücken et al., 2002). *In vivo* labeling experiments revealed that newly translated D1 proteins are inserted into the thylakoid membrane and that the formation of the heterodimeric PSII reaction center, built up by the connection of the pre-D2-Cyt*b*_559_-precomplex (pre-D2) and the pD1-PsbI precomplex (pre-D1) is the first blocked step in PSII biogenesis in *hcf136* mutants. Consequently, this leads to the accumulation of radiolabelled PSII core proteins in the free protein fraction and precomplexes, proving that HCF136 is essentially required for the assembly, stability and/or repair of the heterodimeric PSII RC complex in *Arabidopsis* (Meurer et al., 1998; Plücken et al., 2002).

Interestingly, the removal of YCF48 in *Synechococcus* sp. PCC 7002 did not result in any obvious phenotypical changes and PSII accumulated normally (Shen et al., 2002). In contrast, inactivation of the *YCF48* gene in *Synechocystis* affected PSII and pigmentation but led to milder phenotypical defects as compared to the *Arabidopsis hcf136* mutant (Meurer et al., 1998; Komenda et al., 2008). In contrast to *hcf136* in *Arabidopsis*, the *ycf48* strain was able to grow photoautotrophically albeit its growth was severely slowed as compared to the “wild type strain” (Komenda et al., 2008). At present, it remains unclear if there are discrepancies between different strains and species concerning the function of HCF136/YCF48. However, the function of YCF48 in *Synechococcus* sp. PCC 7002 and presumably other cyanobacteria becomes probably more important under conditions which require fast repair of PSII, such as heat or high light which induce damage of PSII.

*In vivo* labeling analysis revealed that the assembly of the PSII reaction center is also the first step of PSII biogenesis, which is impaired in the *ycf48 Synechocystis* mutant, in line with the observed effects in the corresponding plant mutant. However, the cyanobacterial *ycf48* deletion mutant was still able to assemble higher order PSII complexes, though more slowly, contrasting with their complete absence in the chloroplast (Plücken et al., 2002; Komenda et al., 2008). As a consequence, free PSII core proteins and corresponding precomplexes accumulated in both phylogenetic lineages (Meurer et al., 1998; Plücken et al., 2002; Komenda et al., 2008). Additionally, the absence of YCF48 led to a severe reduction in the levels of the cyanobacterial precursor forms of D1 (pD1 and iD1) and their availability for fast-turnover of D1 after photodamage (Komenda et al., 2008). Native gel analysis showed that the plant HCF136 co-migrated predominantly with PSII precomplexes up to the PSII reaction center, but very little amounts could still be observed in the regions of the RC47 precomplex, the PSII monomer and dimer, whereas YCF48 could only be detected up to the PSII reaction center in cyanobacteria (Plücken et al., 2002; Komenda et al., 2008). In line with these results, an interaction of YCF48 with unassembled pD1 and iD1 could be observed in split ubiquitin assays in cyanobacteria (Komenda et al., 2008). Altogether, these observations strongly suggest that plant HCF136 and its cyanobacterial homolog play a specific role in the stabilization of newly synthesized pD1 and/or subsequent dimerization of pre-D1 and pre-D2 to form the PSII reaction center and are also involved in PSII repair after photoinhibition (Meurer et al., 1998; Plücken et al., 2002; Komenda et al., 2008).

### Interaction Partners of HCF136/YCF48 and Assembly of PSII

HCF136/YCF48 interacts with another PSII assembly factor, namely PAM68/Sll0933 in *Arabidopsis* and *Synechocystis*, respectively (Armbruster et al., 2010; Rengstl et al., 2013). In contrast to the dual localization of YCF48 in cyanobacterial thylakoid membranes and PDMs, Sll0933 resides only in the thylakoid membrane and has been proposed to be relevant for the conversion of the PSII reaction center complex into the RC47 complex and into larger PSII complexes (Rengstl et al., 2011, 2013). Nevertheless, several lines of evidence exist that both proteins interact with each other in an at least transiently formed intermediate PSII complex. In the absence of Sll0933, YCF48 was found to co-migrate with smaller complexes and its distribution shifted toward the PDMs (Rengstl et al., 2011). Moreover, a strong reciprocal dependency between the protein levels of YCF48 and Sll0933 and their reduction in the absence of D1 further reinforce a direct link between the two proteins (Rengstl et al., 2011). YCF48 has also been reported to interact with a Hyper Conserved Protein (PSHCP) of unknown function which was found recently in marine picocyanobacteria. This protein is specific for this clade and conserved to 100% (Whidden et al., 2014). Apart from its interaction with YCF48, PSHCP is also associated to the 50S ribosomal protein L2 and PsaD, pointing to a direct connection of protein synthesis and PS biogenesis via PSHCP (Whidden et al., 2014) and suggesting insertion of thylakoid proteins *in statu nascendi* in *Prochlorococcus* and marine *Synechococcus* species. However, the precise molecular mechanisms underlying PSII assembly, the exact role of HCF136/YCF48 and other PSII assembly factors and the complicated network between them remain elusive to a large part. Current knowledge suggests the following scenario for the early steps of PSII biogenesis: Simultaneously with the co-translational insertion of pD1 into the membrane, it is provided with recycled chlorophyll via the YCF39-Hlip complex in cyanobacteria (Nickelsen and Rengstl, 2013; Chidgey et al., 2014; Knoppová et al., 2014). Afterwards pD1 is loaded with Mn^2+^ by PratA (probably by LPA1 in plants) and builds up the pre-D1 complex together with PsbI (Nickelsen and Rengstl, 2013). HCF136/YCF48 subsequently binds to pD1 in the lumen and assists in the formation of the PSII reaction center by connecting the pre-D1 and pre-D2 complexes (Komenda et al., 2008; Nickelsen and Rengstl, 2013). This step also requires PsbN located on the stromal side in chloroplasts (Torabi et al., 2014). All these early steps of PSII assembly are thought to take place in PDMs in cyanobacteria and in the stroma lamellae in chloroplasts (Danielsson et al., 2006; Rengstl et al., 2011; Nickelsen and Rengstl, 2013). The RC complex connected at least to YCF48 moves subsequently toward the thylakoid membrane where the pre-CP47 complex containing PAM68/Sll0933 waits to be associated (Rengstl et al., 2011). YCF48 and the YCF39-Hlip complex are thought to be released upon the formation of the so-called RC47 complex (Komenda et al., 2008; Knoppová et al., 2014). It is conceivable, that YCF48, Sll0933 and the YCF39-Hlip complex work in a concerted manner together with factors responsible for chlorophyll synthesis to ensure that production of harmful reactive oxygen species is avoided and that pigments are inserted properly into the PSII complex to achieve functional PSII complexes (Rengstl et al., 2011; Chidgey et al., 2014; Knoppová et al., 2014).

## PsbN

### Discovery, Localization and Topology of PsbN

The *psbN* gene was originally identified in liverwort and named ORF43 (Kohchi et al., 1988) before the protein was renamed erroneously as PSII subunit PsbN based on N-terminal sequencing of PSII complex proteins of *Synechococcus vulcanus* (Ikeuchi et al., 1989). It then turned out that the identified N-terminus protein did not belong to PsbN but to PsbTc (Kashino et al., 2002a). Moreover, the PsbN protein could not be found associated to PSII complexes in any proteomic or crystal structure analysis (Gomez et al., 2002; Kashino et al., 2002a,b; Guskov et al., 2009; Plöscher et al., 2009; Umena et al., 2011). This all disputed the assumptions that PsbN is a constituent subunit of PSII.

Only recently, it could be shown, that PsbN does not co-localize with PSII complexes in the grana core but accumulates in the stroma lamella and is – if at all – only transiently associated to other complexes or proteins (Torabi et al., 2014). Taken together, this provides evidence that PsbN is not a constituent subunit of PSII. Instead, a detailed functional characterization of transplastomic Δ*psbN* tobacco mutants revealed its function as the only plastid-encoded PSII assembly factor known so far (Torabi et al., 2014).

PsbN is a conserved plastid encoded low-molecular-weight protein of approximately 5 kDa corresponding to 43 amino acids in most photosynthetic organisms. The N-terminus of PsbN forms an α-helical anchor located in the stroma lamellae. Only a few N-terminal amino acids are expected to extend into the lumen. The stromal exposed C-terminus is predicted to be linked by a flexible spacer who might confer dynamic movability to the C-terminus. Due to its high conservation, the hydrophilic C-terminus is thought to represent the main functional part of PsbN (Torabi et al., 2014).

### Gene Context and Expression of *PsbN*

In most cyanobacteria, the *psbN* gene resides next to *psbH* on the opposite strand and represents one of the few genes that were never integrated successfully into the nuclear genome of any photosynthetic organism during evolution (Mayes and Barber, 1991; Race et al., 1999). Instead, the plastid *psbN* gene is expressed from the strand opposite of the highly conserved *psbB* gene cluster, residing in the intergenic region between *psbTc* and *psbH* of all vascular plants (Stoppel and Meurer, 2013). The transcription of *psbN* depends on the plastid-encoded RNA polymerase (PEP) and is regulated by the sigma factor SIG3 in *Arabidopsis* (Zghidi et al., 2007). Hence, the expression of *psbN* might influence the processing of the *psbT-psbH* intercistronic RNA by enabling double-strand specific cleavage (Chevalier et al., 2015). Additionally, it was hypothesized that read-through transcription of *psbN* potentially could lead to the production of antisense RNA resulting in translational inactivation and protection of the *psbTc* mRNA (Zghidi et al., 2007; Zghidi-Abouzid et al., 2011). The accumulation of *psbN* mRNA was shown to be photoresponsive. However, the effect of light differs between pea and wheat seedlings concerning the abundance of the *psbN* transcript. In pea, *psbN* mRNA was absent in etiolated seedlings but enhanced transcript accumulation occurred upon illumination (Kohchi et al., 1988). In contrast, *psbN* mRNA decreased slightly during greening in wheat seedlings (Kawaguchi et al., 1992). In *Arabidopsis*, PsbN seems to play an important role already during early development, since significant amounts of PsbN protein were detected in etiolated seedlings, which strongly increased after the onset of light much before the appearance of the constituent subunits of the photosynthetic complexes. PsbN levels decreased again after 24 h to reach a constant plateau in the light (Torabi et al., 2014). The tobacco 5′ UTR of *psbN* harbors two processing sites which were reported to be crucial for the translation rate of *psbN* mRNA *in vitro* and therefore could offer an explanation for the *in vivo* fluctuations of *psbN* transcript and protein levels observed in different species (Kuroda and Sugiura, 2014).

### PsbN is Required for Early PSII Assembly and Repair

Three different *psbN* knock-out plants have been generated in *Nicotiana tabacum* via plastid transformation to reveal the function of its gene product. Two *psbN* knock-out plants were generated by inserting a resistance cassette in forward (Δ*psbN*-F) and reverse (Δ*psbN*-R) orientation into the *psbN* gene to estimate potential side effects of the transcriptional direction of the transgene on the transcription of the adjacent genes within the *psbB* operon (Torabi et al., 2014). To overcome possible secondary effects induced by the insertion, a Δ*psbN* mutant was created by a new co-transformation approach replacing the natural *psbN* gene by a mutated *psbN* allele harboring a frameshift mutation and simultaneously integrating a selection marker into a neutral insertion site further away from the *psbB* operon (Krech et al., 2013). All three homoplastomic tobacco mutants were somehow able to grow photoautotrophically but were extremely light sensitive and retarded in growth even when grown on sucrose supplemented medium. The mutants reached maturity solely under very low light intensities (Krech et al., 2013; Torabi et al., 2014) and they were not able to recover from photoinhibition efficiently (Torabi et al., 2014). Spectroscopic measurements revealed that the mutant plants displayed essentially similar deficiencies in terms of severely impaired PSII activity and intactness leading to a diminished electron transport toward PSI (Torabi et al., 2014). The lack of PsbN led to drastic deficiencies of PSII proteins and, presumably as a secondary effect, a slight reduction of PSI protein levels (Krech et al., 2013; Torabi et al., 2014) even though translation of photosynthetic transcripts was not impaired (Torabi et al., 2014). Detailed biochemical analysis showed that the efficient formation of the dimeric PSII reaction center is obviously the first step during PSII complex assembly in which PsbN plays a crucial role. The lack of PsbN protein had no effect on the formation of intermediate precomplexes (pre-D1, pre-D2, pre-CP43 and pre-CP47), these complexes even transiently accumulated in the mutants. The assembly of the dimeric PSII reaction center was, however, very ineffective, leading to a reduced abundance of all higher order PSII complexes (Torabi et al., 2014).

Interestingly, although translation of PSI reaction center proteins were comparable to the wild type, the *psbN* mutants assembled higher order PSI complexes much faster. This very likely represents a secondary effect due to an altered thylakoid structure in Δ*psbN*. In conclusion, PsbN specifically functions as an authentic assembly factor already during early PSII biogenesis and is also involved in the PSII repair cycle (Torabi et al., 2014). Whether PsbN acts on its own or in transient association with other proteins assisting the assembly process remains to be shown.

A *psbH psbN* double mutant in *Synechocystis* did not exhibit a visibly deteriorated phenotype compared to mutant strains lacking solely PsbH (Mayes et al., 1993) suggesting that PsbN is not essential for PSII assembly in this cyanobacterial species. Nevertheless, it is reasonable to assume that the cyanobacterial PsbN fulfills the same or a similar function in PSII assembly as its counterpart in higher plants, in accordance with its highly conserved C-terminus (Torabi et al., 2014). However, a more detailed investigation of a cyanobacterial *psbN* single mutant would be required to definitively determine the function of PsbN in this clade. Especially the lack of low-molecular-weight proteins in cyanobacterial mutants often leads to more relieved phenotypes in comparison with their eukaryotic counterparts, arguing for the presence of compensatory mechanisms and a greater flexibility of cyanobacteria in terms of environmental and genetic modifications in cyanobacteria (Shi and Schröder, 2004). On the other hand, the chloroplast could have evolved a sensitive protein complex quality control system, which includes heat shock proteins, chaperones and the proteolytic action of proteases to remove not properly assembled or destroyed structures (Malnoë et al., 2014). This may explain the dispensability of other PSII assembly factors, such as HCF136 and PsbN, in cyanobacterial species in contrast to the chloroplast system (Meurer et al., 1998; Shen et al., 2002; Komenda et al., 2008).

### PsbN and HCF136 Assist the Formation of the PSII RC on Opposite Sides of the Membrane

**FIGURE 3:**
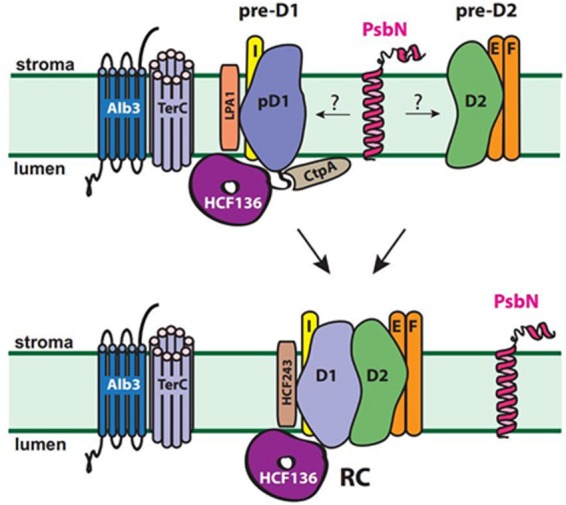
**Role of factors required for early assembly of the heterodimeric PSII reaction center complex in chloroplasts.** Proposed interactions of CtpA, LPA1, HCF243 and HCF136 are shown. The interaction of PsbN with PSII precomplexes and/or other assembly factors remains to be elucidated. HCF136 assists on the lumenal side of the stroma lamellae and presumably the functional C-terminus of PsbN on the stromal side in the heterodimerisation of pre-D1 and pre-D2 to form the PSII reaction center (RC). ALB3 and TerC are required for efficient post- and co-translational insertion of proteins into the PSII complex (see **Figure 4**).

Strikingly, PsbN and HCF136 have many main features in common. For instance, they share their specific function in PSII reaction center assembly, their conservation during evolution and their localization in the loosely packed stroma lamellae (Meurer et al., 1998; Plücken et al., 2002; Torabi et al., 2014). Moreover, different from knock-outs of most other PSII assembly factors, *hcf136* and *psbN* mutant lines both exhibit a severely impaired growth and photosynthetic performance and both proteins exhibit a crucial role already during very early assembly of PSII (Meurer et al., 1998; Torabi et al., 2014). This hypothesis is further supported by their early appearance in etiolated seedlings and similar light–induced expression pattern (Meurer et al., 1998; Torabi et al., 2014). The formation of PSII precomplexes occurs at a normal rate and the PSI complex assembly is even accelerated, whereas the formation of the heterodimeric PSII reaction center is the first step which is impaired in PSII assembly in both mutants (Plücken et al., 2002; Torabi et al., 2014). In contrast to the lumenal localization of HCF136, the conserved and presumably functional C-terminus of PsbN resides on the opposite side of the membrane (**Figure 3**). This renders a direct physical interaction of both proteins rather unlikely (Plücken et al., 2002; Torabi et al., 2014). However, all the shared characteristics indicate that both assembly factors function in a concerted manner to enable the formation of the PSII reaction center. They also demonstrate that factors for the assembly of the PSII complex are required on both sides of the thylakoid membrane.

## TerC

### Discovery and Localization of the TerC Protein

TerC is an ancient gene and most probably originates from prokaryotic ancestors. The nuclear-encoded *Arabidopsis* tellurite resistance C protein (AtTerC) is an integral thylakoid membrane protein with a molecular mass of 37 kDa, which shares its conserved TerC domain with a bacterial gene product, TerC, which is involved in conferring resistance to the highly toxic element tellurium (Jobling and Ritchie, 1987, 1988). An initial study analyzing AtTerC knock-out mutants revealed that the protein is essential for chloroplast biogenesis, since the mutants are highly pigment-deficient and seedling lethal (Kwon and Cho, 2008). Electron microscopy was used to analyse the transition from proplastids to chloroplasts and revealed that AtTerC is indispensable for formation of the prolamellar body prior to light exposure as well as for thylakoid formation after illumination. Consequently, thylakoid membrane complexes are lacking entirely (Kwon and Cho, 2008). Grown under lower light conditions, a minor amount of LHCPs was accumulating and PSII assembly factors, such as ALB3, PAM68, LPA1 and LPA2 were detectable (Schneider et al., 2014). It was speculated that AtTerC might either be involved in the translocation or insertion of thylakoid membrane proteins, the attachment of polysomes to the membrane or even during the arrangement of lipids from the non-bilayer to the bilayer lipid phase (Kwon and Cho, 2008). A recent study utilized an mRNAi knock-down mutant in addition to the null-mutant to further investigate the function of AtTerC (Schneider et al., 2014). Although Kwon and Cho demonstrated that neither the transcription nor association of mRNA with ribosomes is responsible for the lack of accumulation of plastome-encoded proteins, Schneider et al. (2014) could show that the synthesis of PSII subunits is severely affected in knock-out as well as knock-down mutants. Moreover, complementation of the *terc* mutant with a C-terminally GFP-tagged AtTerC fusion protein could only partially rescue the phenotype, indicating an interference with AtTerC function caused by altering the C-terminus of the protein. Intriguingly, AtTerC could interact with ALB3 *in vivo*, suggesting that the two proteins may function in concert during the co-translational insertion of plastome-encoded subunits. Split-ubiquitin assays further showed an additional direct interaction with the PSII subunits D1, D2 and CP43 as well as with LPA1 and PAM68, all together indicating that TerC may function in several steps of PSII assembly, but is especially indispensable during the early formation of the PSII reaction center (Schneider et al., 2014).

### Origin and Proposed Function of TerC in Bacteria

Tellurite-resistance-genes have been identified in *Escherichia coli* and other bacteria, where they are part of the *ter* operon, containing the *terZABCDEF* genes (Anantharaman et al., 2012). However, the exact mode of function of these gene products in tellurite resistance has remained enigmatic over the past decades. In addition to tellurite resistance, some gene products of the *ter* operon also play a role in resistance to other xenobiotics, colicines and bacteriophages. In this respect *terBCD* and *E* seem to be specifically required for tellurite resistance (Taylor et al., 2002), whereas *terDC* and *Z* have a more general function and disrupt all resistance mechanisms (Whelan et al., 1995). Upon contact with tellurite it was observed that black crystals are formed and studies in *Pseudomonas* cells (*P. putida* BS228 and *P. aeruginosa* ML4262) suggested that these are secreted from the cells via vesicle budding from the outer membrane (Suzina et al., 1995). TerC domains from all organisms commonly share 7 transmembrane domains (8 are predicted for AtTerC) (Kwon and Cho, 2008; Anantharaman et al., 2012). In these hydrophobic domains several conserved charged residues are found and it was speculated that these residues could provide an anionic surface within the membrane, thus functioning as a pore or as a binding site for metals (Anantharaman et al., 2012). In some putative bacterial transport proteins, the TerC domain is linked to the CorC_HlyC motif presumably involved in magnesium and/or cobalt efflux (UniProt: Q3A573). The CorC_HlyC motif is also found at the C terminus of some Na^+^/H^+^ antiporters indicating a function of TerC in modulating transport of ion substrates. This is a feature that might also play a crucial role for protein insertion into the thylakoid membrane and it will, thus, be exciting to unravel the exact role of AtTerC during these processes in the future. Since counterparts of any of the other *ter* gene products could not be found in the genome of *Arabidopsis* the function of TerC might have diverged and is not related to ion transport in chloroplasts.

## ALB3

### Characteristics of the YidC/Oxa1/ALB3 Protein Family

ALB3 was initially identified by Sundberg et al. (1997) due to the isolation of an *Arabidopsis* mutant showing an albinotic and seedling lethal phenotype (Sundberg et al., 1997). The ALB3 is a thylakoid-membrane protein with a molecular mass 40–45 kDa and is related to the protein insertases YidC and Oxa1 that are located in the bacterial plasma membrane and the inner membrane of mitochondria, respectively (see Hennon et al., 2015 for a recent review). Together they comprise the YidC/Oxa1/ALB3 protein family, which is more conserved on a structural level than on a primary sequence level and most representatives possess five membrane-spanning regions (Saller et al., 2012). Specificity their substrate recruitment is most likely conferred by their varying C-termini, which differ in length and structure and thus provide the prerequisite for an either co- or post-translational mode of action. Both YidC and Oxa1 are necessary for the insertion of protein components of the respiratory complexes, whereas ALB3 substrates are thylakoid-membrane proteins. Notably, bacterial, chloroplast and mitochondrial proteins were frequently observed to functionally complement each other, thus suggesting a widely conserved insertion mechanism (Jiang et al., 2002; Preuss et al., 2005; Benz et al., 2009). Recently, the first crystal structure of YidC from *Bacillus halodurans* could be solved in which the five transmembrane regions were found to form a positively charged hydrophilic cavity, which is closed from the extracellular side of the membrane, but opening to the cytoplasm and the lipid bilayer (Kumazaki et al., 2014).

### SRP-Mediated Post-translational LHCP Insertion Mechanism

Following the initial identification and characterization of the *alb3* mutant in *Arabidopsis*, a model proposing a function of ALB3 in the membrane insertion of light-harvesting complex proteins (LHCPs) was rapidly established (Moore et al., 2000; Woolhead et al., 2001). LHCPs associated with both, PSI and PSII, are exclusively encoded in the nucleus and need to be post-translationally targeted to the chloroplast and the thylakoid membrane. Hence, the chloroplast has gained a unique signal recognition particle (SRP) system, involving the chloroplast specific cpSRP43 and the GTPase cpSRP54, the latter which, although it is evolutionary conserved, lacks the typical RNA component (Schünemann et al., 1998). After import and processing of the LHCP precursors a conserved DPLG motif in the third transmembrane helix of the LHCPs binds to an ankyrin repeat in cpSRP43. Together with cpSRP54, which in turn interacts with chromodomains of cpSRP43, a transit-complex is formed (Hermkes et al., 2006; Stengel et al., 2008). To target this soluble transit-complex to the thylakoid membrane a receptor protein is required, which is represented by cpFtsY, a protein related to the bacterial homolog of the SRP receptor, FtsY. The LHCPs are thought to be transferred to ALB3 prior to their insertion since cpFtsY was shown to directly interact with ALB3 (Moore et al., 2003; Asakura et al., 2008). Moreover, an association of cpSRP43 and ALB3 has been observed and investigated in further detail (Bals et al., 2010; Falk et al., 2010; Falk and Sinning, 2010; Lewis et al., 2010; Dünschede et al., 2011). ALB3 exposes its extended intrinsically disordered C-terminal domain into the stroma, which adopts an alpha helical fold upon interaction with cpSRP43 (Falk et al., 2010). The C-terminus comprises four positively charged motifs (I–IV), and two models for the interaction with cpSRP43 have been suggested. On the one hand, motifs II and IV were identified as important binding determinates (Falk et al., 2010). On the other hand, Dünschede et al. (2011) suggested a model of ALB3 forming a dimeric pore with motif II and a membrane embedded region being involved in cpSRP binding (Dünschede et al., 2011). Moreover, a mutant expressing a truncated ALB3 protein lacking motifs III and IV did not show a significant effect in LHCP accumulation, although the C-terminus seems to be important for ALB3 stability in a light-dependent manner (Urbischek et al., 2015). Possibly, cpSRP43 is initially captured by an interaction with motif IV, but the insertion into the membrane requires other domains of ALB3 and the exact mechanism remains to be elucidated.

### SEC-Mediated Post-translational LHCP Insertion Mechanism

In contrast to this unique SRP-mediated post-translational LHCP insertion mechanism, the related proteins YidC and Oxa1 are also involved in co-translational protein insertion. YidC can act in concert with the SecY translocon in bacteria, and Oxa1 interacts with ribosomes, thus integrating mitochondrial encoded proteins in a co-translational manner. Considering the severe albinotic phenotype of *ALB3* mutants and the reduced levels of all thylakoid membrane complexes in the mutant, an essential role in co-translational protein insertion of ALB3 is feasible. Chloroplasts contain an evolutionary conserved, but minimalized Sec system, which is suggested to transport proteins post-translationally into the thylakoid lumen (Albiniak et al., 2012). However, cpSecY also interacts with chloroplast ribosomes, was found in the vicinity of D1 elongation intermediates and cpSecY maize mutants display a severe loss of thylakoid membranes (Roy and Barkan, 1998; Zhang et al., 2001). Although LHCP insertion was shown to be independent of cpSecY, it could be demonstrated that at least a portion of ALB3 is associated with cpSecY (Klostermann et al., 2002; Benz et al., 2009).

### Interaction Partners of ALB3

**FIGURE 4:**
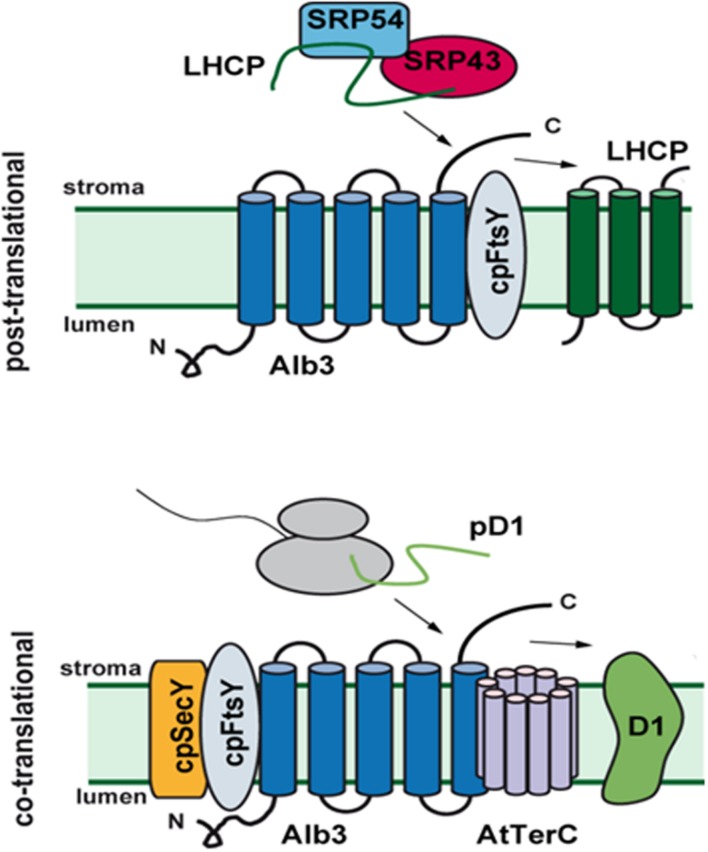
**Role of ALB3 and AtTerC in protein insertion into the thylakoid membrane.** ALB3 functions in the insertion of the nuclear-encoded LHCPs, which are post-translationally targeted by the soluble SRP transit-complex **(upper)**. Co-translational integration of chloroplast-encoded proteins is also mediated by ALB3, which thereby acts in concert with TerC, cpSecY and cpFtsY **(lower)**.

More recently, D1 insertion intermediates could be co-precipitated with ALB3, FtsY, cpSRP54 and Vipp1 (Walter et al., 2015a). Nevertheless, cpSRP54 mutants display only a mild phenotype, arguing against a prominent role of cpSRP54 in co-translational D1 insertion (Amin et al., 1999; Walter et al., 2015b). FtsY, in contrast, seems to be more important since severe defects especially during the PSII repair cycle were observed in the cpFtsY mutant (Walter et al., 2015b). In addition, split-ubiquitin assays showed an interaction of ALB3 with the chloroplast-encoded PSII subunits D1, D2, CP43 as well as with the PSI subunit PsaA and the ATP synthase subunit CF_O_-III, strengthening a role of ALB3 in the co-translational insertion of several chloroplast-encoded proteins, in addition to D1 (Pasch et al., 2005). Moreover, D1 synthesis and PSII complex assembly were found to depend on ALB3 protein levels, albeit being independent of motifs III and IV in the C-terminal domain (Urbischek et al., 2015). The co-translational mechanism is likely to be corroborated by a number of auxiliary factors in addition to ALB3 and interestingly ALB3 has been found to interact with a number of proteins involved in the biogenesis of PSII, such as LPA2 and LPA3 and TerC (see below) (Ma et al., 2007; Cai et al., 2010; Schneider et al., 2014) (**Figure 4**). Future studies will be required to understand their mode of function on a molecular level.

### Conserved Function and Homologs of ALB3

In addition to ALB3 in *Arabidopsis*, studies in the green algae *Chlamydomonas reinhardtii* and the cyanobacterium *Synechocystis* have indicated a function of ALB3 in co-translational D1 insertion. In *Chlamydomonas reinhardtii* two ALB3 orthologs are found, ALB3.1 and ALB3.2. Both are found in one complex and depletion of ALB3.1 results in a clear impairment in LHCP accumulation. However, an RNAi strain of ALB3.2 showed impaired biogenesis of PSI and PSII, suggesting complementary functions for the two orthologs in post- and co-translational protein insertion (Bellafiore et al., 2002; Ossenbühl et al., 2004; Göhre et al., 2006). The *Synechocystis* encodes only for a single ALB3 ortholog, designated Srl1471. Equipment of Srl1471 with a C-terminal tag resulted in a reduced D1 insertion into the membrane and a lower assembly into PSII complexes in the Srl1471 mutant (Spence et al., 2004; Ossenbühl et al., 2006).

In *Arabidopsis*, a second homolog of the YidC/ALB3/Oxa1 family has been identified in chloroplasts (Gerdes et al., 2006). In contrast to ALB3, ALB4 mutants only show a mild phenotype with retarded growth. ALB4 was shown to play a role in CF_1_-CF_O_-ATP synthase assembly and stabilization (Benz et al., 2009). Although ALB4 lacks the C-terminal extension, recent studies analyzing double mutants suggest that ALB4 participates in the ALB3-mediated protein insertion pathway possibly restricted to specific substrates, such as Cyt *f* and the Rieske protein (Trösch et al., 2015).

## Outlook and Summary

The inner core of PSII contains more than 20 structural proteins which bind all cofactors and are able to perform light absorption, electron transfer and oxygen evolution. To date more than 30 auxiliary proteins have been identified (see recent reviews; Shi et al., 2012; Nickelsen and Rengstl, 2013; Järvi et al., 2015) and many more are to be expected by avant-garde biochemical and genetic approaches, allowing to discern between secondary and primary effects in corresponding mutants. This means that the auxiliary proteins of the PSII complex are outnumbering the structural proteins by a factor of two to three. It will be an extremely challenging task to elucidate how these auxiliary proteins keep PSII in an optimal state to efficiently perform photosynthesis under changing environmental conditions. Of special interest will be to understand if these auxiliary proteins function individually in series or in assembled complexes in a concerted way and how they are coordinated in order to promote PSII function. Future studies will elucidate whether they form dynamic complexes which change their compositions according to the requirement of the plant cell. Furthermore, the sequential attachment of most of the intriguingly numerous low-molecular-mass subunits remains to be solved. Moreover, almost nothing is known regarding the incorporation of cofactors and the protein factors involved in those processes. To understand the function and to unravel the complicated network of auxiliary proteins will be a major task for scientists in the field of photosynthesis for the next decade.

## Author Contributions

All authors listed, have made substantial, direct and intellectual contribution to the work, and approved it for publication.

## Conflict of Interest Statement

The authors declare that the research was conducted in the absence of any commercial or financial relationships that could be construed as a potential conflict of interest.

## Abbreviations

Cyt: cytochrome
PSII: photosystem II
